# The changing epidemiology of Japanese encephalitis and New data: the implications for New recommendations for Japanese encephalitis vaccine

**DOI:** 10.1186/s40794-017-0057-x

**Published:** 2017-08-01

**Authors:** Bradley Connor, William B. Bunn

**Affiliations:** 1 The New York Center for Travel and Tropical Medicine, Weill Medical College of Cornell University, 110 East 55th Street, 16th Floor, New York, NY 10022 USA; 2 Medical University of South Carolina, University of Illinois at Chicago School of Public Health, Chicago, USA

**Keywords:** Japanese encephalitis, Japanese encephalitis vaccine, Flaviviruses, Peri-urban risk, Changing epidemiology, Mosquito transmittal diseases, Travel vaccines, Underappreciated risks, ACIP, Accelerated dosing of vaccines

## Abstract

The epidemiology of Japanese Encephalitis and risk to the traveler has changed and continues to evolve. The spread of Japanese Encephalitis virus into new environments, changes in agricultural practice and animal vectors, climate change, peri-urban growth, changes in international travel to Asia, personal risk factors, mosquito vector free transmission, interactions with other flaviviruses and better information on infections without encephalitis and other factors make Japanese Encephalitis an underappreciated risk. There has also been a change in the incidence of Japanese Encephalitis cases that questions the current travel duration and geographic based recommendations. A safe, effective vaccine (Ixiaro) that may be administered in a short course regimen is now available in the United States without the risks of the previous vaccine. However, the vaccine is significantly underutilized. These changes in the epidemiology and new data on the risks of the Japanese Encephalitis virus require a review of the practice guidelines and expert recommendations that do not reflect the current state of knowledge.

Japanese Encephalitis virus (JEV) is a mosquito borne single standard RNA virus related to West Nile flavivirus and is the most common cause of vaccine preventable encephalitis in Asia and a large area of the western Pacific including northern portions of Australia. The vector of Japanese Encephalitis (JE) transmission is primarily through the bite of Culex species mosquitoes (primarily Culex tritaeniorhychus). The virus is maintained and amplified by intermediate hosts, primarily pigs and wading birds. Transmission occurs most frequently in agricultural areas such as farms and rice paddies; however transmission also occurs in peri-urban or urban areas with appropriate ecologic conditions [1].

In endemic countries JE is primarily a disease of children since adults have acquired immunity through previous infection. Of individuals infected less than 1% will progress to encephalitis [2]. Among the 30,000 – 50,000 global cases per year approximately 20 – 30% of patients will die and 30 – 50% of survivors will have significant neurologic or psychiatric sequelae [3]. The incubation period is 5 – 51 days and symptoms begin with headache, fever, vomiting and progress to mental status changes, neurologic deficits, and movement disorders. Seizures are common especially in children. JE cannot be distinguished from other encephalopathies by clinical presentation, laboratory testing is needed. There is no specific antiviral treatment, only supportive care and management of complications. The occurrence of JE is seasonal in temperate climates with the primarily period of risk from May through October; however, in tropical regions the risk is year round. The greatest diurnal risk is the biting period of the mosquito during the crepuscular period (dusk) until dawn [3–5].

The epidemiology of JE and risk to the traveler has changed and continues to evolve. The spread of JEV into new environments, changes in agricultural practice and animal victors, climate change, peri-urban growth, changes in international travel to Asia, personal risk factors, mosquito vector free transmission, interactions with other flaviviruses and better information on infections without encephalitis and other factors make JE an underappreciated risk. There has also been change in the incidence of JE cases that questions the current time and geographic based recommendations. A safe, effective vaccine that may be administered in a short course regimen is now available in the United States without the risks of the previous vaccine. However, the vaccine is significantly underutilized. These changes in the epidemiology and new data on the risks of the JEV require a review of the practice guidelines and expert recommendations which currently do not reflect the current state of knowledge. Most important of these are the recommendations of the Advisory Committee on Immunization Practice (ACIP) of the United States Centers for Disease Control and Prevention (CDC) [2, 4, 6].

## Geographic change

A significant change in the global risk of JE has been a major geographic expansion in Asia. Almost half of the world’s population now lives in a country where JE is endemic. International travel to Asia has significantly increased over the last two decades and United States multinational corporations have significantly increased Asian operations [5, 6].

## Agricultural changes

The transmission of JE is dependent on the transmission to humans by mosquitoes and the amplification in pigs and birds. Pig rearing and the industrialization of pig farming with dense herds have increased significantly in many areas. In China, pork production doubled from 1990 to 2005[2] and the areas of pork production have spread due to urbanization. In cities poultry and pigs are preferred for urban agriculture. Studies have shown significant numbers of Aedes species mosquitoes in cities with increased numbers in areas with animal/pig keeping. The risks are also increased with rice farming which has significantly increased in production acreage. The production acreage has also varied by country and within countries [5].

## Ecologic factors

The ecologic and geographic factors for JEV are not clearly understood. The emergence of JEV in Tibet, a cold high altitude region or the development of two new serotypes in Australia shows the spread of JEV is unpredictable. Land cover and land use also has a significant impact in JEV spread [5].

## Birds

Birds are known amplifying and reservoir hosts. Many species are widely distributed and migratory birds and have been associated with spread from country to country and with the geographic increase in endemic areas. The spread of JE from birds will increase with the increase in urban husbandry of birds and the reports of urban mosquitoes carrying JEV increase risk in urban areas. Concern for area spread of JEV through birds into new regions including North America has been studied [6–8].

## Climate change

Global climate change can have a significant impact due to temperature changes and rising sea levels in coastal urbanized areas. A number of mosquitoes are salinity tolerant including vectors of JE (Culex tritaeniorhynchus) [9] and may increase with rising sea levels. Temperature increases may cause a change in the peak and duration of the transmission season and an increased risk particularly in temperate climates [8–10].

## Changing risks from rural exposure

The increased risk of suburban or peri-urban areas has been documented in multiple Asian countries (South Korea, China, Singapore, Taiwan) [5, 6]. A recent study of JE cases in South Korea showed a significant shift from rural to suburban or at the periphery of urban areas. The relative risk of JE infection in urban and suburban areas of South Korea was similar to other areas [8, 11]. This shift suggests that JE should not be considered only a disease of rural or farming areas. The increasing exposure in peri-urban or suburban settings questions the thought that the risk is limited to rural areas [5, 8, 11].

## Changing profile of international travelers

Historically and in many recommendations, adventure and leisure travelers were considered the only travelers with significant JE risk. Current patterns have called these assumptions into question. Business travel and international assignments have been growing with the globalization of large corporations. Multinational corporations (MNCs) have expanded outside their home countries not only to produce goods at lower costs for home consumption but also to expand overseas markets particularly in Asia. For many MNCs, international revenues already exceed domestic revenues [12, 13]. Expatriates represent a key category of International Business Travelers. Expatriates and commonly their families will live and work in the host country and require access to quality health care during the assignment. The number of countries with expatriates or assignees also continues to increase. Currently, MNCs place international expatriates or assignees in more than 20 countries on average, and the number of countries continues to grow. For MNCs, 95% of the consumers are outside the United States, and the growth in revenues from developing countries will drive continued globalization and the need for business travel and overseas assignments [12–14].

Business travelers’ health and safety risks have historically been considered “low”. However, studies have shown that these risks are similar to the risks of other international travelers [14, 15]. In fact, in a recent study of travelers to Asia, more than 60% of high risk travelers listed a business reason for the trips [16]. Studies have also shown more hospitalizations and evacuations for “low risk” travelers, although the country risk level was also a predictor [13].

## Peri-urban risk

A significant shift in JE risk for business travelers is the local destination of business travelers. There is a significant shift from urban business meetings and hotels to peri-urban exposure. MNCs now increasingly manufacture in high risk countries and the trend continues in Asia. The manufacturing and housing facilities are often located many miles from the major cities. For example the location of expatriate and guest housing for a major United States MNC near a pig farm near Beijing highlighted the risk [6]. With increased manufacturing facilities the number of international visitors and assignees and expatriates has increased. International travelers and assignees provide engineering, financial, auditing and training support. The destination of international business travelers is now more commonly the manufacturing site in peri-urban or rural areas. Many business visitors perform extensive outside work such as engineers and environmental site auditors [17].

## Personal factors and Japanese encephalitis risk change

Personal risk factors may increase the risk associated with the Japanese Encephalitis. Older age is an important risk factor for clinical illness with the risk of neuro-invasive illness five to tenfold higher in people aged 50 and older compared with older children and young adults. Younger age is also a risk factor for symptomatic illness and is associated with a higher frequency of neurologic sequelae. There also may be an increased risk with pregnancy [6].

The demographics of international travelers are changing. The number of the older adult travelers from the United States has increased and continues to increase. In addition, there are increasing numbers of immuno incompetent travelers with better treatment for cancer and infectious disease [6].

## Non-mosquito vector transmission in pigs

There has been a recent interest in non- vector transmission of flaviviruses. A recent article described vector free transmission of JEV in pigs [18, 19]. JEV infection in pigs and birds is associated with amplification of the virus which was then communicated to other animals and humans by mosquitoes. However, investigation of some JEV outbreaks showed no local mosquitoes with the virus. In an experimental study by Ricklin, “Japanese Encephalitis virus tropism in experimentally infected pigs”, pigs were infected by needle with JEV. The pigs developed a fever that lasted 3–5 days and a viremia of 3 days duration with a decrease in appetite and activity. Although the clinical signs were mild and no neurological changes were observed, all the animals were affected by non-supportive meningoencephalitis myelitis which lasted until day 11 post injection. A striking observation was in the tonsils where the viral loads were high and the JEV appeared to continue to replicate for at least 11 days after the end of the viremia and the viral loads were 100–1000 times higher than other organs. The infected animals shed live virus 6–10 days after injection. A second study showed transmission from infected pigs to uninfected pigs who were housed with the infected pigs. The symptoms and RNA quantities in the various organs were similar between needle injected and sentinel pigs infected by contact. The second study showed persistent viremia in the tonsils at 25 days. The two studies showed that pigs shed JEV in oronasal secretions and can be infected by the oro-nasal route [18, 19].

## Incidence of Japanese encephalitis infections in febrile travelers

A 2016 study of 619 febrile travelers arriving in the port of Shenzhen, China showed 34 cases of dengue virus and 17 cases of JE infections by serum testing with few cases of other mosquito borne diseases (Chikungunya,1, malaria,3, yellow fever virus,O, West Nile virus,O). The study concludes dengue and JE viruses are the primary pathogens of viral infections found in febrile travelers to Shenzhen China ports and that increased monitoring is now needed for these mosquito borne infections [20].

The percentage of JEV positive febrile travelers was striking in this study, particularly given the government sponsored vaccination programs in many Asian countries. Dengue and JE are both flaviviruses and have similar mosquito vectors although they have different transmission cycles. The incidence of dengue has increased dramatically in recent decades [21]. This study does not allow direct comparisons of the incidence of the infections, but it suggests that the incidence of JE infections could also be increasing [20].

## Japanese encephalitis virus infection without encephalitis

The risk of Japanese Encephalitis may be low, but infection and mild or subclinical infections are much more common. The risk of JE infection is approximately 100 times the number of reported JE cases [2]. Although about (1%) of the infected develop encephalitis, another 10% develop minor illness [22]. The recent recognition of the risks of subclinical or mild illnesses with the Zika virus and birth defects have brought forward the question of vaccination to prevent infection of flaviviruses without significant clinical illness. There is also a question of the JE and Zika virus impact on dengue infections although more research is needed [18].

These changes in the epidemiology of flaviviruses and the importance a symptomatic or mild clinical illness question the very low risk statements for JE. The risk of infection and mild illness is much higher and should be considered in travelers to high risk areas [6, 20].

## Changes in trip duration in Japanese encephalitis cases

Recent case studies of JE are informative. There were 55 United States cases published by Hills from 1973–2008. Sixty five percent spent greater than one month in endemic areas. The age range was 1–91 with a median age of 34 [23]. A 2009 review of JE cases globally 1992–2008 found that at least half the cases published or reported were in travelers of less than thirty days [24]. An update in US international travelers through 2014 includes 87 cases and only 50% had visits of over thirty days [6]. More recent cases included a 10-day stopover in Bali in a Swedish traveler, a short visit with little rural exposure in Thailand in a Finnish traveler, a French traveler to Thailand with little rural exposure (only daytime) outside transmission season and, an Australian vacationer to Bali resorts for nine days [6].

### Japanese encephalitis vaccines in the united states

Japanese Encephalitis Vaccine derived from mouse brain tissue was first used in the 1930s. A Vero cell derived vaccine was licensed by the United States FDA in 2009 (JE-VC). The original mouse brain vaccine was reported to have frequent and often severe side effects and US availability ended in 2012 [25–27].

Currently, the only United States licensed JE-Vaccine is a JE-VC vaccine (Ixiaro) [27]. However, other vaccines are available globally [28]. The current recommendation is two doses at 0 and 28 days. It was licensed for 18 years of age and above in 2009 and for greater than two months of age in 2013. (Adult dose is 0.5 mL and pediatric dose is 0.25 mL) In a study by Dubishar-Kastner, if only one dose is given the protection is limited with 43% seroconversion at 28 days. With two doses (0.28) seroconversion is 83% at six months and 48% at one year. With a booster dose at 11, 15 or 23 months the seroconversion is 99% at two years. If the patient was “primed” with an earlier dose of JE-MB the sero protection was 95-98% with single booster dose and 100% if primed with 2 or 3 doses of JE-MB. The safety profile of JE-VC 36 months post vaccine launch was in the expected range with primarily injection site and general flu like illnesses reported. Five years of data (over 1,000,000 doses distributed) now support the safety of the vaccine in the US [6, 25].

A recent accelerated dosing study design (1,7 days) v49-23 Phase 3 observer blinded randomized multicenter study of 661 adults was conducted [29]. The study was also designed to look at compatibility with rabies vaccine which may be given on a similar schedule. The study showed 99% seroconversion at day 15 (7 days after second injection and conversion rates remained high after one year). There was no interference with the concomitant dosing with rabies vaccine or either vaccine. Adverse events were similar in the accelerated and conventional dosing groups. Injection site tenderness was the most common event in both groups with similar numbers of events. Both the standard schedule and accelerated dosing schedule require a booster after one year and require three doses but accelerated dosing should allow more coverage [29].

### Japanese encephalitis vaccine utilization

The utilization of JE vaccine in United States travelers with travel to endemic/Asian countries has been low. In a recent study of the Global TravEpiNet clinics, a sophisticated consortium of expert traveler medicine providers only 26.8% of travelers deemed by 15 current ACIP recommendations to be at high risk received JE vaccine. (4% of lower risk group) [16]. Previous studies of travelers to Asia and airport questionnaires showed 1% to 11% vaccination rates [17, 30]. The primary reason that the vaccine was not given in the Global Epi Net study was “the vaccine was not indicated (55%)” despite meeting ACIP recommendations [16, 25]. Another reason for failure to vaccinate (16.6%) was inadequate time for the second doses (28 days) before travel [17]. The new accelerated dosing schedule will significantly reduce the failure to vaccinate due to the time of the trip departure.

### The need for New recommendations

JE is a changing and unpredictable threat for travelers and a safe effective vaccine is available. The recent epidemiologic studies and new data suggest that the risks of JE are changing and increasing globally. The case studies show that multiple travelers with short visits not meeting the current ACIP recommendations of “a month or longer in endemic areas during the travel season” have contracted the disease. The case studies also show that the disease occurs in tourist areas and in peri-urban areas or near large cities (eg., Bejing) and cases continue to occur. Epidemics are unpredictable and cases, particularly mild disease, are underreported and risks are understated. The utilization of JE vaccines is poor primarily due to failure to understand or follow recommendations [16, 25].

The current ACIP recommendations for adult travelers have not had meaningful changes since 1993 when only mouse brain vaccine, which had frequent and often severe side effects, was the only vaccine available. Minimal changes to the ACIP recommendations were made in 2010 but failed to take into account the current state of our knowledge of the changing epidemiology of risk. Since 2009, a safe and effective vaccine has been available and since 2012 has been the only vaccine available in the United States. The current recommendations need revision for travelers and long-term residents or assignees [6].

A Japanese Encephalitis Working Group met in conjunction with the American Society of Tropical Medicine and Hygiene Meeting in November 2014 and reconvened at the meeting in November 2015 and 2016. The Group conclusions included: The current recommendations are narrowly-focused. JE vaccine is only “recommended” for travelers who plan to “spend a month or longer in endemic areas during the transmission season”. The criterion of one month is arbitrary. There have been deaths with short visits. In fact, the “high risk” category only captures half of the reported JE travel-related cases since 1973 [23, 24]. The risk of contracting JE should be addressed in an individual discussion of risks with a health care provider. The current recommendation also states that risks are in “rural or agricultural areas”. However, in 2014 there were large numbers of travelers (e.g. business travelers, researchers) to peri-urban areas where there were clear risks for JE [16]. Furthermore, although duration of travel (such as > 1 month) does play a role, such durations have been eliminated from the recommendations for other travel vaccines such as rabies and typhoid, as it is clear that exposure can occur at any time, even during shorter trips [6, 25].

The current recommendations also include a number of groups for “consideration” of vaccination. In the “consideration” category for short-term travelers, a number of “at risk” activities are listed including (“camping, hiking, trekking, biking, fishing, hunting or farming”). This description is largely based on leisure travelers [2]. Travel patterns particularly to Asia have changed. Business travelers, researchers and humanitarian travelers now may make up 60% of at risk travelers from the United States [16]. Each traveler’s activities and risks should be fully discussed with a health care provider [6, 25].

Updated clean and concise ACIP recommendations for JE vaccine are needed to reflect current and changing “at risk” areas and address the needs for all travelers through a provider-patient discussion.

## Recommendations for Japanese encephalitis vaccine

Discussion of JE and availability of a safe and effective vaccine with all travelers to Asia (endemic areas). Travelers to rural or peri-urban areas in endemic countries – irrespective of duration or of travel or itinerary. All expatriates living in endemic countries or frequent travelers who may visit rural or peri-urban areas in endemic countries. Travelers with uncertain itineraries or itineraries that may change.

## Not recommended

Not recommended if travel is restricted exclusively to urban areas.

## Conclusion

In summary, this paper addresses a number of epidemiologic changes that impact the risks of infection with the Japanese Encephalitis virus and then discusses the implications of a number of these studies for the recommendations of the ACIP. The findings suggest that the risk of contracting the virus are growing and are underappreciated. The latter sections discuss the failure of the current vaccination programs to appropriately protect international travelers and the reasons for this failing. The most important reason is that the vaccine was not indicated showing that the ACIP recommendations are not clear. New, clear and concise guidelines are needed and are proposed.

## Abbreviations

ACIP: Advisory committee on immunization practice
CDC: Centers for disease control and prevention
GTEN: Global travEpiNet clinics
JE – MB: Japanese encephalitis vaccine – mouse brain
JE – VC: Japanese encephalitis vaccine – vero cell
JE: Japanese encephalitis
JEV: Japanese encephalitis Virus
MNC: Multinational corporation
